# Transport and Anaesthesia Consideration for Transcatheter Patent Ductus Arteriosus Closure in Premature Infants

**DOI:** 10.3390/jcdd10090377

**Published:** 2023-09-01

**Authors:** Tuan Chen Aw, Belinda Chan, Yogen Singh

**Affiliations:** 1 Department of Anaesthesia, Royal Brompton Hospital, London SW3 6NP, UK; 2 Neonatology Division, Department of Pediatrics, University of Utah, Salt Lake City, UT 84108, USA; belinda.chan@hsc.utah.edu; 3 Department of Radiology and Imaging Science, University of Utah, Salt Lake City, UT 84108, USA; 4 Department of Pediatrics, Division of Neonatology, Loma Linda University School of Medicine, Loma Linda, CA 92354, USA; yogen.singh@nhs.net; 5 Department of Pediatrics, Division of Neonatology, University of Southern California (USC), Los Angeles, CA 92354, USA

**Keywords:** patent ductus arteriosus, closure, transcatheter PDA closure (TCPC), premature babies, transcatheter, devices, surgery, transport, anaesthesia

## Abstract

Transcatheter device closure of patent ductus arteriosus (PDA) in preterm infants has been proven to be a feasible and safe technique with promising results when compared to surgical ligation. However, managing transport and anaesthesia in extremely premature infants with haemodynamically significant PDA and limited reserves presents unique challenges. This review article focuses on the key considerations throughout the clinical pathway for the PDA device closure, including referral hospital consultation, patient selection, intra- and inter-hospital transport, and anaesthesia management. The key elements encompass comprehensive patient assessment, meticulous airway management, optimised ventilation strategies, precise thermoregulation, patient-tailored sedation protocols, vigilant haemodynamic monitoring, and safe transport measures throughout the pre-operative, intra-operative, and post-operative phases. A multidisciplinary approach enhances the chances of procedure success, improves patient outcomes, and minimises the risk of complications.

## 1. Introduction

A persistent patent ductus arteriosus (PDA) is common in extremely low-birth-weight gestational infants (ELGAN) under 28 weeks of gestational age (GA), with a higher incidence in lower GA [1]. Transcatheter PDA closure (TCPC) has emerged as a promising and safe therapeutic option for infants with haemodynamically significant PDA (hsPDA) who have failed pharmacological closure or have contraindications to medical therapy, and is gradually replacing surgical PDA ligation in most infants [2].

The recent approval of the Amplatzer Piccolo Occluder in 2019 for PDA device closure in infants weighing over 700 g and older than 3 days of life has increased the use of TCPC in ELGAN infants, and now, in select centres, it has become the preferred method over surgical ligation [3]. TCPC has demonstrated lower mortality and co-morbidities, and shorter hospital stay in comparison to surgical PDA ligation, avoiding thoracotomy and other invasive surgical risks [3].

Providing TCPC for ELGAN infants needs special expertise such as an interventional cardiologist with expertise in performing TCPC and a team to support the whole procedure care pathway. Hence, it poses significant challenges to provide this therapy widely, due to limited expertise and available specialised centres. Many cases are referred from outside hospitals, presenting difficulties in patient referral and neonatal transport [4]. Even for centres and physicians with extensive experience in paediatric catheterisation, performing TCPC in infants weighing less than 1 kg remains an emerging procedure [5]. The Royal Brompton Hospital, London, UK (RBH), for example, performs 750–850 paediatric transcatheter procedures a year but only a fraction (about 10–15 cases per year) involve infants weighing under 1500 g; the intricacies of performing TCPC in extremely premature infants with haemodynamic instability and limited reserves demand expertise and specialised care.

To establish a successful ELGAN TCPC program, learning from centres with established clinical pathways is important. This article highlights the main differences in transport and anaesthesia management of ELGAN infants undergoing TCPC and provides guidance on overcoming the challenges.

## 2. Referral Process Considerations

### 2.1. Patient Selection

Transporting ELGAN infants to specialised centres for TCPC presents substantial challenges that necessitate meticulous planning [4]. Before proceeding with the transport, the foremost importance is selecting the ideal TCPC candidate with a thorough assessment and discussion with the specialist paediatric cardiology centres. Table 1 provides the clinical criteria for determining the eligibility of ELGAN infants for TCPC [3]. Table 2 lists the echocardiography findings commonly used to diagnose hsPDA [6].

### 2.2. Multidisciplinary Team Consultation

A multidisciplinary team consultation is needed for transferring patients from the referral hospital to the specialised centre for TCPC. The referring neonatal team consults the accepting paediatric cardiology team to evaluate the clinical information and discuss the need for PDA treatment. If the criteria for TCPC are met (Table 1 and Table 2), the cardiac interventionalist is contacted to schedule the procedure, or the case is discussed in the joint cardiology–cardiothoracic (JCC) meeting to agree upon the best intervention—TCPC or surgical ligation. Clear communication between the referring and accepting healthcare professionals through a telemedicine conference is recommended and this multidisciplinary team meeting should involve the referring neonatologist, accepting intensivist/neonatologist, paediatric cardiologist, and cardiac interventionalist/cardiothoracic surgeon when possible. The patient should be transferred to the specialist centre the day before TCPC has been scheduled [4]. The referring hospital should be promptly notified of any altered plan, even after admission to the accepting hospital.

### 2.3. Parental Communication

Parents should actively participate in the shared decision making and informed consent process. The accepting hospital may be more than 300 miles (or 480 km) away [4], which adds stress for parents. Parents should be informed about the inherent risks associated with transport and TCPC procedures, especially for ELGAN infants. The clinical condition of a preterm infant with hsPDA can change rapidly, and parents should understand that treatment plan may be modified after assessment at the specialist cardiology centre. The procedure may be delayed or cancelled, especially if the infant becomes unstable after transport or other emergency cases take priority.

## 3. Inter-Hospital Transport Considerations

Transporting ELGAN infants, particularly those with hsPDA or haemodynamic instability, requires a specialised team understanding of the specific cardiovascular physiology. A neonatal expert transport team, consisting of a neonatologist, a neonatal nurse, and a respiratory therapist (in the USA and Canada), can more effectively monitor and manage vulnerable ELGAN infants [4,7]. Transportation can be conducted via land, air, or a combination of both. The selection of transport modes depends on staff availability, travel distance, weather conditions, and the patient’s clinical condition [4]. In general, ambulance transport is suitable for distances less than 100 miles (160 km) [4]. For longer distances, helicopter or rotary flight can reduce travel time. Fixed-wing aircraft are more appropriate for distances exceeding 300 miles [4].

All transport modes subject the ELGAN infants to various environmental stressors, including ambient noise, sensory stimuli, vibration, handling, acceleration and deceleration forces, temperature fluctuations, limited space for emergencies, inadequate monitoring during transit, suboptimal ventilatory support, unforeseen traffic accidents, and altitude effects in unpressurized helicopters [4,7]. Greene et al. reviewed 620 neonatal transport cases in different settings, travel distances, and transport modes. They found that 25–92% of infants had abnormal vital signs including oxygen saturation, heart rate, temperature, and systolic blood pressure. Hypothermia was observed in 15% of neonates during transport, especially among preterm infants and longer transport distance [8]. These factors can increase the risk of intraventricular haemorrhage and compromise cerebral perfusion in ELGAN infants who have limited cerebral autoregulation capabilities [7]. Advanced equipment, such as a well-designed transport incubator, neonatal-specific ventilator, thermal mattress, and neurodevelopmental head positional support, can help mitigate these risks [4].

Intrinsically, ELGAN infants may experience respiratory decompensation, haemodynamic instability, hypothermia, hypoglycaemia, and airway-related issues during transport [9]. ELGAN infants requiring TCPC have limited haemodynamic reserves and are particularly susceptible to pulmonary over-circulation and respiratory decompensation. Uniquely with PDA, supplemental oxygen administration may decrease pulmonary vascular resistance and lead to increased left-to-right shunting through the ductus arteriosus, thus exacerbating hypoxia and systemic hypoperfusion. Transport-related issues may arise that are unforeseen in routine NICU care. For example, internal air expands when atmospheric pressure decreases in higher altitudes. The oropharyngeal tube can decompress the enlarged stomach with expanded air, thus preventing compressing on the diaphragm. ELGAN infants are at an increased risk of developing pulmonary interstitial emphysema, a condition characterized by over-distended alveoli. As the air expands in higher altitudes, those alveoli may further enlarge and lead to pneumothorax, or existing pneumothorax aggravates during air transport [10]. The limited space in the transport vehicle makes it hard to perform thoracocentesis; similarly, it is hard to perform intubation if the endotracheal tube is dislodged, or to replace venous access. A neonatal transport clinician is crucial in optimizing ventilation settings throughout the journey. Upon arrival at the accepting hospital, proper handover and communication are essential. With meticulous preparation, experienced transport teams can safely transport most infants weighing under 1 kg without complications [4].

## 4. Anaesthesia Considerations

Anaesthesia management for ELGAN infants undergoing TCPC involves significant differences throughout the pre-operative, intra-operative, and post-operative phases. The anaesthesia team faces various challenges related to the complexity of medically fragile ELGAN infants, the unique layout of the procedural room, the use of uncommon equipment, thermoregulation, vascular access, sedation, and managing cardiorespiratory instability. The anaesthetist overseeing these challenges should thoroughly understand the catheter laboratory environment, be skilled in handling neonatal airway equipment, have knowledge of their specific ventilator capabilities, and be proficient in ultrasound-guided vascular access. We will discuss specific strategies for the anaesthesia team to ensure patient preparation, maintain stability during the procedure, and facilitate a seamless post-procedure recovery.

### 4.1. During the Pre-Operative Phase

The anaesthesia team manages (a) patient pre-assessment, (b) clinical optimisation, (c) procedure location and equipment, and (d) safe patient transport.

#### 4.1.1. Patient Pre-Assessment

**Figure 1:**
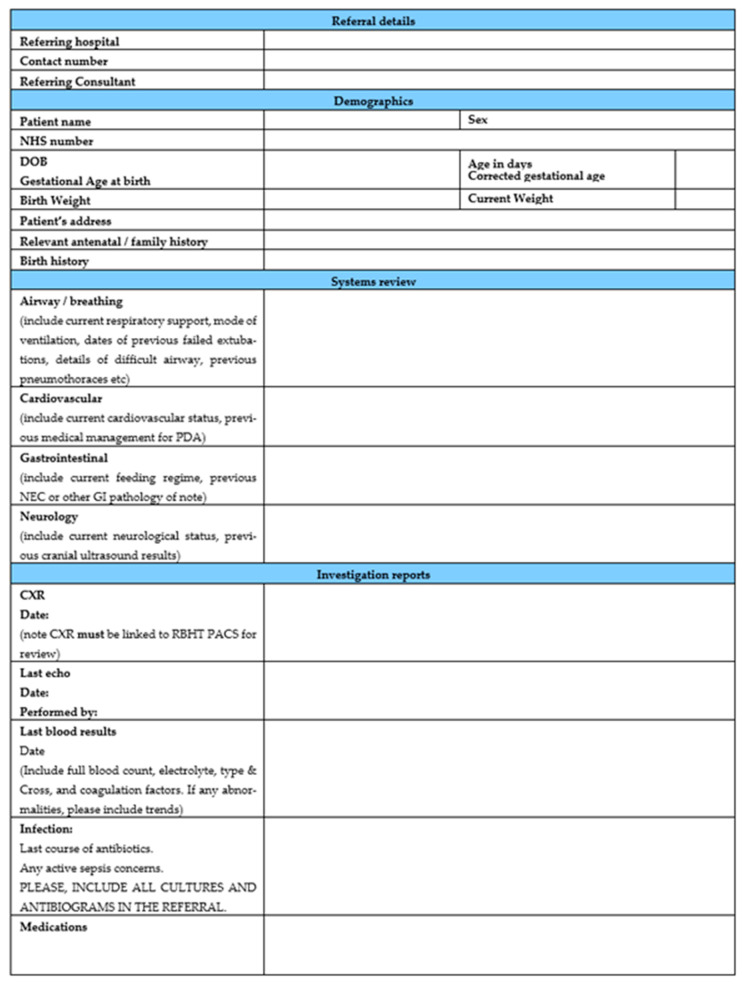
Royal Brompton Hospital PDA referral form.

The complex clinical history and risk of multi-system comorbidities of premature infants with hsPDA necessitate a thorough pre-operative evaluation. Access to an up-to-date proforma with essential patient clinical information completed by the referring team is invaluable for the structured clinical pathway. Table 3 discusses the elements that should be in the patient pre-assessment. Figure 1 shows an example of local proforma used at the Royal Brompton Hospital. This standardized tool facilitates communication between the referring and anaesthesia teams.

#### 4.1.2. Clinical Optimisation

Arranging for blood products, antibiotics, intravenous access, and/or heparin is necessary. Blood transfusion is more often needed than anticipated, making pre-ordering blood products helpful. When audited at RBH, periprocedural transfusion (from during the procedure until 48 h after) in 165 patients showed that 25.5% (*n* = 42) cases required red cell transfusion, 11.5% (*n* = 19) required red cells during the procedure, 3.6% (*n* = 6) platelet transfusion, and 1.2% (*n* = 2) fresh frozen plasma transfusion. One in four patients need a blood transfusion, making pre-emptive blood sampling for cross-matching of red cells a necessary step. Antibiotic prophylaxis may be routine for some centres, and this should be preferably administered in the NICU before transfer to the Cath Lab. ELGAN infants have miniature and fragile vessels—establishing peripheral intravenous access (IV) needs expertise and may take a longer time. Hence, it is recommended to have two reliably working peripheral IVs for maintenance fluids (for glycaemic control), with another venous access for sedation and bolus medication. Most facilities avoid heparin bolus in ELGAN infants, and only use small heparinised flushes to prevent catheter thrombosis [5]. If heparinization is used, it is necessary to double-check the dosage, as errors may occur easily when dealing with an extremely small volume (usually 50–100 u/kg).

#### 4.1.3. Procedure Location and Equipment

##### Choosing the Right Location

TCPC has been successfully performed at the patient’s bedside and in the cardiac catheterisation suite (Cath Lab) following a comprehensive evaluation of feasibility and safety [11]. Even short-distance transport from the NICU to the Cath Lab has inherent risks [11]. Performing TCPC at the bedside is feasible using portable C-arm fluoroscopy and echocardiography, eliminating the need to transport fragile ELGAN infants to the Cath Lab [11]. However, most hospitals prefer to perform TCPC in the Cath Lab due to its numerous advantages. The Cath Lab provides a familiar setting for the anaesthetic and interventional cardiology team, and also offers high-quality fluoroscopy, a large space for equipment, and necessary catheterisation supplies [11].

##### Determining the Cath Lab Utilisation

Adequate time allocation in the Cath Lab is also important. Based on the RBH Cath Lab audit, the median duration of 165 TCPC procedures performed in the Cath Lab was 138 min (IQR 115–165 min), with a minimum of 55 min. These durations align with other facilities reporting median Cath Lab time of 109–121 min, procedure time of 28–32 min, and fluoroscopy time of 4–6 min [12,13]. Therefore, allocating a total case time of up to 3 h is reasonable, considering both the procedure time and transport time between the NICU and the Cath Lab.

##### Space, Layout, Ventilators

**Figure 2:**
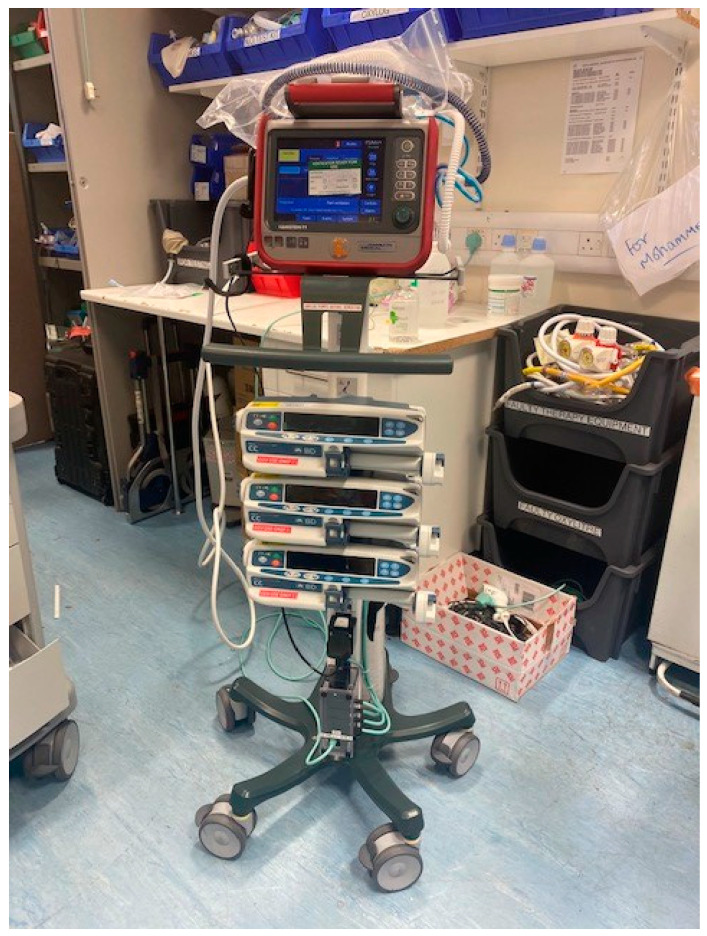
Hamilton-T1 transport ventilator with customized adaptions.

**Figure 3:**
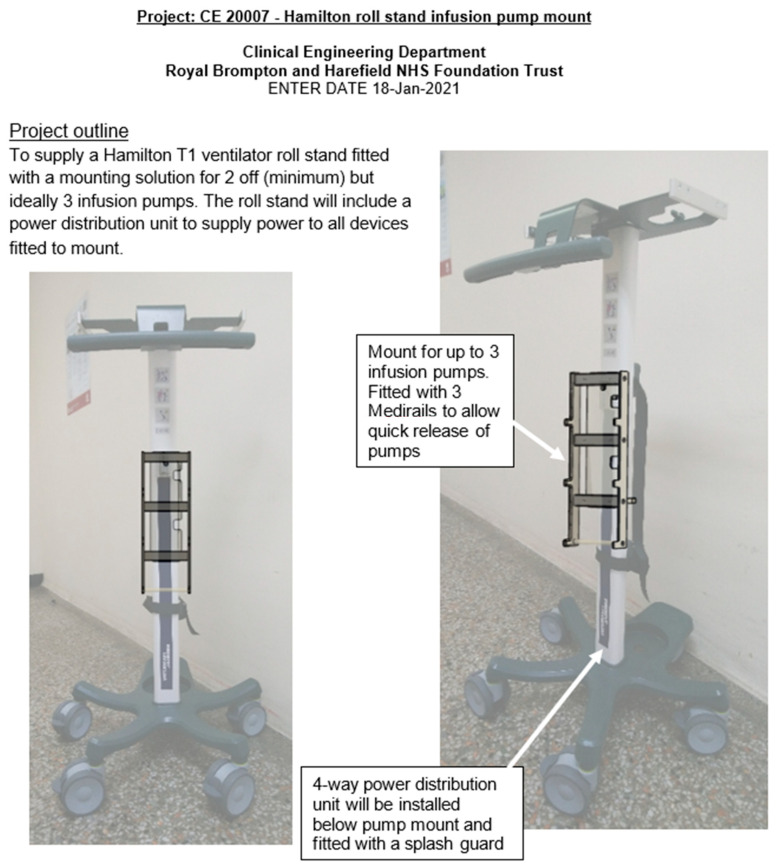
Medirail mount designed for Hamilton ventilator stand (1).

**Figure 4:**
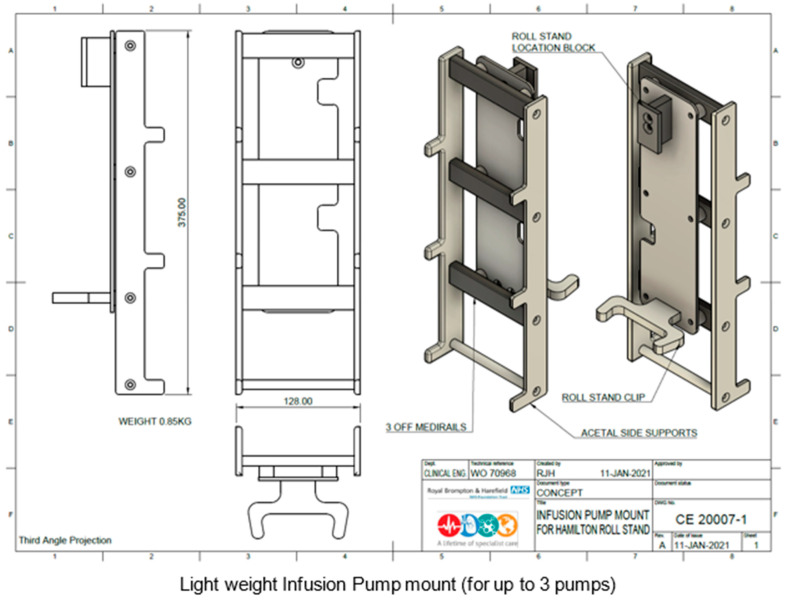
Medirail mount designed for Hamilton ventilator stand (2).

**Figure 5:**
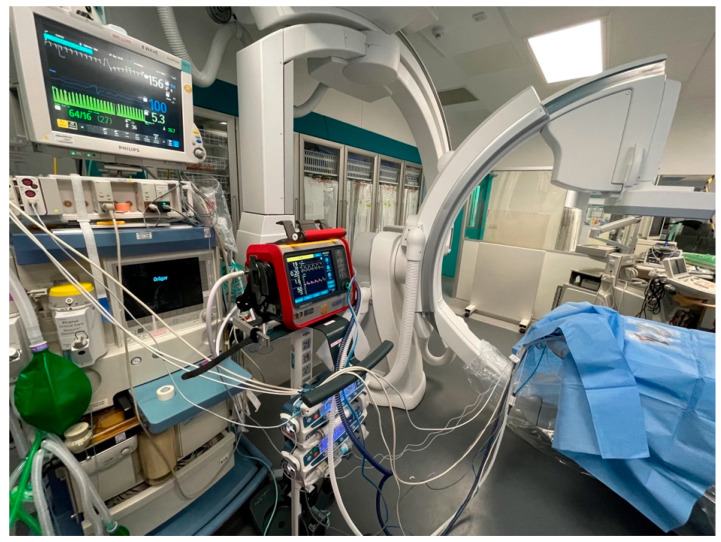
Hamilton transport ventilator in catheter lab position.

Surprisingly, a small ELGAN infant disproportionally needs substantial space in the Cath Lab to accommodate neonatal-specific equipment and a ventilator suitable for neonates. The reason for using a non-anaesthetic ventilator deserves a separate discussion (please see below). The various Cath Lab configurations to accommodate neonatal-specific ventilators consist of: (a) physical space around the procedure table, (b) the position of the base of the primary A-plane arm in relation to the head of the table, and (c) B-plane positioning, which could make using bulkier ventilators along with a syringe driver stand complicated or even unfeasible. RBH Cath Lab has created a creative solution with the current setup of using the Hamilton-T1 transport ventilator with a 3 m ventilation circuit mounted on a Hamilton portable stand. This portable stand has been modified to hold up to three syringe drivers. All the syringe drivers and the transport monitor are powered through a single power outlet. This ventilator unit is slim and portable, supports infusion pumps, and only requires a single power cable and single oxygen connection for use (Figure 2, Figure 3, Figure 4 and Figure 5). It is easily adaptable for any type of catheter lab configuration.

##### Monitoring Equipment

Besides the ventilator setup mentioned above, TCPC requires additional monitoring equipment, namely, two pulse oximetry monitors (pre- and post-ductal), electrocardiogram, non-invasive blood pressure, end-tidal CO_2_, and oesophageal temperature probe. The oesophageal temperature probe also serves as a fluoroscopy landmark for the anterior wall of the aorta. ELGAN infants are sensitive to cerebral perfusion changes, so cerebral near-infrared spectrometry monitoring may be helpful. Adjunct thermoregulation gear is necessary to maintain the infant’s temperature.

Overall, consideration for procedure location and equipment is to balance monitoring and efficient anaesthesia techniques with minimal facility utilisation time and avoiding equipment clutter.

#### 4.1.4. Safe Patient Transport

The final step in the pre-operative phase is the safe transport of the patient from the NICU to the Cath Lab. Like inter-hospital transfer, even short-distance transfers of ELGAN infants can be labour-intensive with risks of accidental extubation, disruption of ventilation, hypothermia, and loss of vascular access that disrupts medication infusions. [4]. Like inter-hospital facility transfer, airway management, ventilation, and thermoregulation are top concerns. If the infant is intubated, endotracheal tube (ETT) can be easily dislodged or malposed in such a small airway. Brief ventilation with a transporting ventilator or T-piece resuscitator can be tolerated [4]. Instead of using the transport incubator, the patient’s closed-lid isolate should be sufficient. It is important to be prepared with backup equipment and an emergency kit, even for short-distance moves [4].

### 4.2. During the Intra-Operative Phase

Thorough pre-operative planning minimises complications and facilitates a smooth transition into the intra-operative phase. Anaesthesia considerations encompass various aspects, namely, (a) respiratory care, (b) induction and maintenance, (c) hypothermia mitigation, (d) period of instability, and (e) procedural emergency preparedness.

#### 4.2.1. Respiratory Care (Airway, Ventilation, and Oxygenation)

##### Airway Management

**Figure 6:**
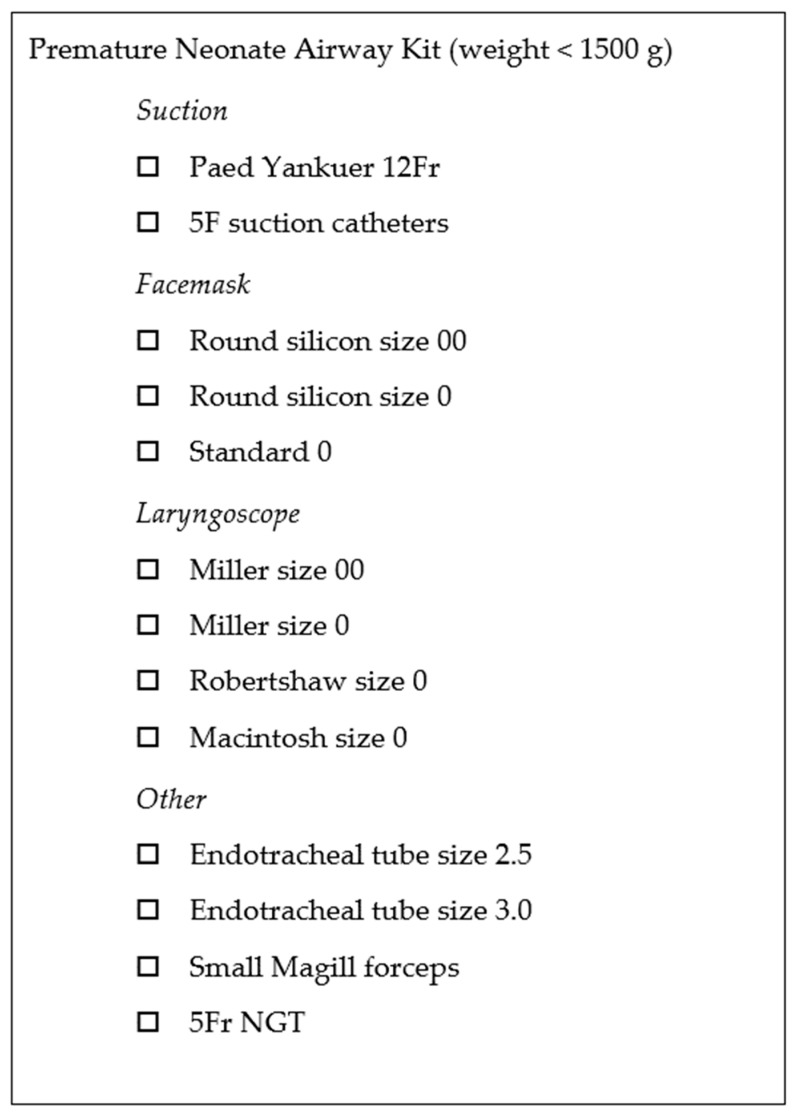
Premature Neonate Airway Pack checklist.

ELGAN infants may require intubation before transport to the Cath Lab if intubation has not been achieved [5]. Re-intubation readiness should also be checked before the patient arrives at the Cath Lab. A complete set of the infant’s airway and intubation equipment must be available and pre-checked. A regularly stocked ‘Preterm Airway Pack’ has been useful (Figure 6). The ETT position could be checked using fluoroscopy at the start of the procedure, and images saved for relevant referencing. The tube should be repositioned immediately if the ETT tip position is suboptimal. ETT tip position should then be fluoroscopically re-checked before proceeding with the catheter intervention. Anticipate other common airway-related incidents, such as kinking, disconnection, ETT tip migration, and secretion-related obstruction.

##### Ventilation Optimisation

Severe respiratory failure is an indication of TCPC, and, along with poorly compliant premature lungs and pulmonary over-circulation from PDA, makes respiratory care in ELGAN infants especially challenging [2]. Achieving optimal ventilation goals while protecting the developing lungs means tight CO_2_ control while avoiding barotrauma, volutrauma, and atelectasis. Wheeler et al. warn about decreased functional residual capacity (FRC) due to atelectasis and ventilation–perfusion mismatch during anaesthetic induction [2]. Standard anaesthetic machines may not adequately meet these goals, necessitating neonatal-specific ventilator use in the Cath Lab. Therefore, it is essential to pre-emptively assess the Cath Lab layout and space to accommodate these non-anaesthetic ventilators, as discussed earlier. RBH uses the Hamilton-T1 ventilator for its conventional ventilator mode and portable convenience. Some ELGAN infants are in the NICU on the High-Frequency Oscillatory Ventilator (HFOV) or High-Frequency Jet Ventilator (HFJV). A study has shown that these ventilators can be used successfully in the Cath Lab for TCPC [12]. Other authors had suggested a trial off HFOV/HFJV and onto a conventional ventilator for the ease of transport the day before the procedure, only if the patient tolerates it [4]. Careful deliberation is necessary when choosing between conventional and high-frequency ventilators and determining the specific ventilatory strategies during TCPC [2]. Without clinical trials to support the choice of ventilators, the decision would be based on the patient’s clinical status and institutional experience [2]. Arterial and central venous access is not routinely available for blood gas monitoring, so instead, end-tidal CO_2_ monitoring or transcutaneous CO_2_ monitoring should be used for tight ventilation management. Venous blood gas could be available during at least two time points. One is the time when TCPC catheter venous access is established. Another time would be at the end of the procedure before removing the venous sheath.

##### Oxygenation

Desaturation is common in ELGAN infants, and it is often addressed with increasing supplemental oxygen during hypoxia episodes or giving unnecessary pre-oxygenation before the procedure. However, one should be aware that oxygen is a potent pulmonary vasodilator that exacerbates pulmonary overcalculation. Excessive use of 100% fractional oxygen increases the risk of retinopathy of prematurity and oxygen-free radical damage to end organs like the brain, lungs, etc. [14]. Goal oxygen saturation should be based on unit protocol and gestational age; for example, goal Oxygen Saturation for infants ≤ 32 weeks GA was 89–93%, increasing to >91–95% after 32 weeks GA. Optimisation of positive end-expiratory pressure on the ventilator and avoiding atelectasis should also be considered [2]. 

#### 4.2.2. Induction and Maintenance

Some patients may be well sedated with opioid infusion before the Cath Lab. For those requiring intubation and anaesthesia induction, fentanyl and/or ketamine can be used, followed by any paralytic agent of choice. Slow inhalational induction with sevoflurane may sometimes be necessary if peripheral venous access is deemed unreliable at induction. This, however, is a carefully measured process of balancing pulmonary and systemic circulation, especially in the presence of PDA, to facilitate vascular access, paralytic administration, and intubation if needed. After establishing a secure airway, IV infusion for sedation and ventilation using the non-anaesthetic ventilator of choice should commence. Sedation maintenance can be achieved with low-dose fentanyl, and a paralytic agent if needed.

#### 4.2.3. Hypothermia Management and Mitigating the Risk of Hypothermia

Extremely premature neonates are vulnerable to developing hypothermia, especially in the operating room or Cath Lab. Their high surface area to body weight ratio and lack of insulating fat layer predisposes to rapid falls in core temperature. An undesirable consequence of significant hypothermia includes a risk of pulmonary hypertension, hypoperfusion of vital organs, hypoglycaemia, and metabolic acidosis. Despite this knowledge, mitigating hypothermia, especially for procedures conducted outside the NICU, is still very challenging. Success requires aforethought and concerted effort from all team members. A ‘temperature care bundle’ strategy described by Alhalabi et al. has proven successful in this effort [15], and important points are listed in Table 4.

#### 4.2.4. Period of Instability

There can be expected periods of desaturations and potential cardiovascular instability during this procedure, most notably when the delivery sheath is in the arterial duct and during echocardiography. The delivery sheath can splint open the tricuspid valve, leading to increased tricuspid regurgitation when the catheter restricts the main pulmonary artery flow [5]. Desaturation can be more profound in the presence of a patent foramen ovale or atrial septal defect due to exacerbated right-to-left intra-cardiac shunting [5]. Echocardiography during this time can also restrict chest wall compliance when the echo probe compresses upon the chest wall [10]. Despite expected desaturations during these epochs, it is still prudent to simultaneously check and exclude the common airway issues mentioned above. Low diastolic blood pressure is associated with hsPDA from significant left-to-right shunting [16]. It is important to ensure adequate diastolic blood pressure for coronary perfusion. Measures such as avoiding supplemental oxygen, minimising anaesthesia concentration, and increasing systemic vascular resistance with metaraminol or phenylephrine can be considered [16].

#### 4.2.5. Procedural Emergency Preparedness

Table 5 lists the potential complications and emergencies associated with TCPC. Prevention and management strategies from the anaesthesia team’s perspective are suggested [17,18].

### 4.3. During the Post-Operative Phase

Patients ventilated in NICU should remain sedated and ventilated after the procedure. Immediate extubation in the Cath Lab back to non-invasive ventilation can be considered for patients who were on non-invasive ventilation. We have not found early extubation to be a risk factor for device embolisation. Local anaesthesia with 0.8 mL/kg 0.25% Levobupivacaine at the access site will help for post-procedural comfort for extubated patients. Acute renal injury from IV contrast exposure is a possible but rare post-operative complication, warranting urine output and creatinine monitor [17,18]. Post-ligation cardiac syndrome (PLCS) is a condition that can occur after PDA closure, characterised by a sudden increase in systemic vascular resistance and a decrease in left ventricular preload. There would be an increase in pulmonary venous pressure and cardiorespiratory decompensation, particularly in patients with an immature myocardium. PLCS and respiratory decompensation is less likely to be associated with TCPC than with surgical PDA ligation (18 vs. 78%, *p* < 0.001) [19]. Follow-up echocardiography should be performed within the first 2–6 h after TCPC to evaluate for any post-procedural complications and to assess the position of the PDA closure device [5]. This helps ensure the success of the procedure and detects potential issues earlier. Overall, careful monitoring and appropriate post-procedural management are essential to optimise outcomes and ensure the well-being of patients undergoing TCPC. Patients can usually be safely transferred back to the referral hospital a day after the procedure [4].

## 5. Future Directions

**Figure 7:**
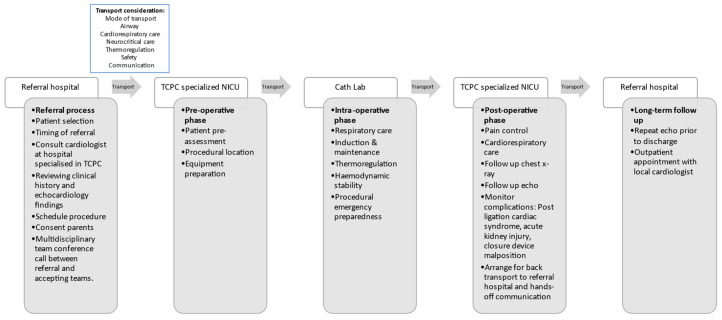
Flow diagram of the transport and anaesthesia process.

Future directions in TCPC for ELGAN infants include addressing knowledge gaps and exploring innovative approaches. Research should focus on accurately identifying infants eligible for TCPC, both locally and at specialised centres. Minimising patient movement by safely performing bedside TCPC using echocardiography alone without fluoroscopy should be explored. Human factors and system engineering approach would help implement transport and anaesthesia-related quality-improvement projects balancing patient safety and performance efficiency of TCPC (Figure 7). A well-designed, multi-centre quality-improvement project is needed to evaluate how process change can affect neonatal outcomes.

## 6. Summary

The increasing need for TCPC in ELGAN infants relies on a robust referral program, safe transportation, and effective anaesthesia management. A well-established process ensures proper patient selection and safe inter-hospital transport. The anaesthesia team serves a vital role and works collaboratively with other healthcare professionals to achieve a successful TCPC program. Centres aiming to pilot an ELGAN TCPC program can learn and pair with more established TCPC centres and senior anaesthetists.

## Figures and Tables

**Table 1:** Clinical criteria for Transcatheter PDA closure [3].

Inclusion Criteria
Current weight >700 g Postnatal age at least more than 3 days of life, preferably 7–30 days after birth Echocardiographic evidence of an hsPDA Clinical symptoms indicating an hsPDA, mechanical ventilation, or persistent inreased respiratory/oxygen support are the most common indicators Failed at least one or two courses of pharmacological treatment (indomethacin, ibuprofen, or paracetamol (acetaminophen)), or when pharmacological treatment is contraindicated Stable for transport on assessment by neonatal transport team
**Exclusion Criteria**
Active sepsis Ongoing treatment for necrotising colitis Ductal dependent congenital heart disease Pulmonary hypertension, unless it is secondary to PDA

**Table 2:** Echocardiography findings to diagnose hemodynamically significant PDA [6].

PDA Characteristics
Size (>1.5 mm for GA ≤ 26 weeks; ≥2.0 mm for GA ≤ 30 weeks) Left to right shunt Pulsatile flow pattern
**Evidence of Pulmonary Over-Circulation**
Left atrium to aorta ratio (≥1.6) Increased left ventricular end diastolic diameter (LVEDD)—Z-score > 2SD Presence of mitral valve regurgitation Left pulmonary artery (LPA) end diastole flow (>40 cm/s) Reversed mitral valve E/A ratio (>1) Left ventricular output (>300 mL/kg/min)
**Systemic Hypoperfusion**
Retrograde blood flow in descending aorta Retrograde or absent end-organ blood flow in the middle cerebral artery, renal artery, coeliac artery, or superior mesenteric artery (absence/reverse diastole flow)

**Table 3:** Pre-operative assessment.

Patient Pre-Assessment
Current clinical condition and stability
2. Deficits in clinical optimisation
3. Significant comorbidities
4. A complete and thorough echocardiography at the accepting hospital, including details on: a. PDA size and morphological type b. Degree of pulmonary hypertension c. Presence of patent foramen ovale or atrial septal defect
5. Chest X-ray a. Check the endotracheal tube position (if present) b. Assess lung pathology
6. Ventilator settings
7. Intravenous access
8. Current medication and infusions and allergies a. Maintenance fluids and glycaemic control b. Sedation and analgesia c. Inotropes
9. Relevant laboratory results
10. Type and Cross-matched blood
11. History of positive culture and antibiotic sensitivity to guide surgical prophylaxis

**Table 4:** Good-practice items for hypothermia mitigation [15].

Thermoregulation Elements
Pre-emptive heating of the procedure room temperature, between 22 and 26 degrees Centigrade (72–78 degrees Fahrenheit) as the World Health Organization recommends for the delivery room Pre-heated forced air mattress Perform intubation and peripheral IV access in the NICU, better equipped for thermoregulation and routine neonatal procedures Limit body exposure at all times and use plastic drapes to cover Use oesophageal temperature probe as able Pre-warm echocardiography gel and immediately wipe off from the patient after scanning

**Table 5:** Potential TCPC-specific intra-operative complications [17,18].

Complication	Incidence	Description	Prevention and Management
Cardiac perforation	1.3%	*Catheterisation instrument punctures right atrium, right ventricular outflow tract, or ventricle* *Causes pericardial effusion or cardiac tamponade*	*Blood transfusion, fluid resuscitation, vasopressor, and/or inotrope for hypovolaemia and hypotension* *Pericardiocentesis and/or surgery*
Vascular injury	1%	*Catheterisation instruments injure peripheral (e.g., femoral vein) or central vessels (e.g., inferior vena cava)* *Causes bleeding, haematoma, or thrombus*	*Blood transfusion, fluid resuscitation, vasopressor, and/or inotrope for hypovolaemia shock* *Provide haemostasis and correct coagulopathy* *Monitor for ischemia injury or thrombus formation* *Utilise ultrasound or fluoroscopy to guide catheter advancement*
Pulmonary artery obstruction	2%	*Device embolisation, migration, or protrusion into the main or branch pulmonary artery* *Causes acute desaturation from pulmonary artery obstruction*	*Symptoms depend upon the degree of obstruction* *Provide cardiovascular and pulmonary instability support during device retrieval or surgical removal* *Echocardiography and fluoroscopy to ensure proper device position and fitting*
Aorta obstruction	2%	*Device embolisation, migration, or protrusion into the descending aorta* *Causes hypoperfusion to the legs, intestines, kidneys, or other vital organs*	*Symptoms depend on the degree of obstruction* *Provide cardiovascular support during device retrieval or surgical removal* *Echocardiography and fluoroscopy to ensure proper device position and fitting* *Post-ductal saturation monitor on the left leg can help to monitor for any sign of descending aorta obstruction* *Oesophageal temperature probe monitor can serve as a landmark for the anterior wall of aorta*
Tricuspid valve injury	2–5%	*Catheterisation instruments injure the tricuspid valve, leading to tricuspid valve insufficiency/regurgitation* *Causes hypoxia from decreased right ventricular outflow, or right-to-left shunting at atrial level*	*Decrease pulmonary vascular resistance with oxygen or inhaled nitric oxide* *Enhance right ventricular function with inotropes like milrinone and dopamine*

## References

[1] Semberova J., Sirc J., Miletin J., Kucera J., Berka I., Sebkova S., O’sullivan S., Franklin O., Stranak Z. (2017). Spontaneous Closure of Patent Ductus Arteriosus in Infants ≤1500 g. Pediatrics.

[2] Wheeler C.R., Vogel E.R., Cusano M.A., Friedman K.G., Callahan R., Porras D., Ibla J.C., Levy P.T. (2022). Definitive Closure of the Patent Ductus Arteriosus in Preterm Infants and Subsequent Short-Term Respiratory Outcomes. Respir. Care.

[3] Kuntz M.T., Staffa S.J., Graham D., Faraoni D., Levy P., DiNardo J., Maschietto N., Nasr V.G. (2022). Trend and Outcomes for Surgical Versus Transcatheter Patent Ductus Arteriosus Closure in Neonates and Infants at US Children’s Hospitals. J. Am. Heart Assoc..

[4] Willis A., Pereiras L., Head T., Dupuis G., Sessums J., Corder G., Graves K., Tipton J., Sathanandam S. (2019). Transport of extremely low birth weight neonates for persistent ductus arteriosus closure in the catheterization lab. Congenit. Heart Dis..

[5] Sathanandam S., Agrawal H., Chilakala S., Johnson J., Allen K., Knott-Craig C., Waller B.R., Philip R. (2019). Can transcatheter PDA closure be performed in neonates ≤1000 grams? The Memphis experience. Congenit. Heart Dis..

[6] Singh Y., Fraisse A., Erdeve O., Atasay B. (2020). Echocardiographic Diagnosis and Hemodynamic Evaluation of Patent Ductus Arteriosus in Extremely Low Gestational Age Newborn (ELGAN) Infants. Front. Pediatr..

[7] Gupta N., Shipley L., Goel N., Carmo K.B., Leslie A., Sharkey D. (2019). Neurocritical care of high-risk infants during inter-hospital transport. Acta Paediatr..

[8] Chang A.S., Berry A., Jones L.J., Sivasangari S. (2015). Specialist teams for neonatal transport to neonatal intensive care units for prevention of morbidity and mortality. Cochrane Database Syst. Rev..

[9] Greene N.D., Riley T., Mastroianni R., Billimoria Z.C., Enquobahrie D.A., Baker C., Gray M.M., Umoren R.A. (2022). Neonatal Vital Sign Trajectories and Risk Factors During Transport Within a Regional Care Network. Air Med. J..

[10] Steenhoff T.C., Siddiqui D.I., Zohn S.F. (2022). EMS Air Medical Transport. StatPearls.

[11] Pouldar T.M., Wong R., Almeida-Jones M., Zahn E., Lubin L. (2021). Bedside Transcatheter Patent Ductus Arteriosus Device Occlusion in an Extremely Low Birth Weight Neonate: A Novel Approach in a High-Risk Population. Case Rep. Anesthesiol..

[12] Shibbani K., Nijres B.M., McLennan D., Bischoff A.R., Giesinger R., McNamara P.J., Klein J., Windsor J., Aldoss O. (2022). Feasibility, Safety, and Short-Term Outcomes of Transcatheter Patent Ductus Arteriosus Closure in Premature Infants on High-Frequency Jet Ventilation. J. Am. Heart Assoc..

[13] Hubbard R., Edmonds K., Rydalch E., Pawelek O., Griffin E., Gautam N. (2020). Anesthetic management of catheter-based patent ductus arteriosus closure in neonates weighing <3 kg: A Retrospective Observational Study. Paediatr Anaesth..

[14] Torr C., Yoder B., Beachy J. (2019). Improving preoperative identification of infants at risk for severe retinopathy of prematurity. J. Perinatol..

[15] Alhalabi E., Zestos M., Kobayashi D., Mckelvey G.M., Taylor R.A. (2022). Interventions to prevent hypothermia in extremely preterm low-weight infants undergoing cardiac catheterisation. BMJ Open Qual..

[16] Rios D.R., Bhattacharya S., Levy P.T., McNamara P.J. (2018). Circulatory Insufficiency and Hypotension Related to the Ductus Arteriosus in Neonates. Front. Pediatr..

[17] Bischoff A.R., Jasani B., Sathanandam S.K., Backes C., Weisz D.E., McNamara P.J. (2021). Percutaneous Closure of Patent Ductus Arteriosus in Infants 1.5 kg or Less: A Meta-Analysis. J. Pediatr..

[18] Sathanandam S., Gutfinger D., Morray B., Berman D., Gillespie M., Forbes T., Johnson J.N., Garg R., Malekzadeh-Milani S., Fraisse A. (2021). Consensus Guidelines for the Prevention and Management of Periprocedural Complications of Transcatheter Patent Ductus Arteriosus Closure with the Amplatzer Piccolo Occluder in Extremely Low Birth Weight Infants. Pediatr. Cardiol..

[19] Sathanandam S., Balduf K., Chilakala S., Washington K., Allen K., Knott-Craig C., Waller B.R., Philip R. (2018). Role of Transcatheter patent ductus arteriosus closure in extremely low birth weight infants. Catheter. Cardiovasc. Interv..

